# Genome-wide identification and transcript profiles of walnut *heat stress transcription factor* involved in abiotic stress

**DOI:** 10.1186/s12864-020-06879-2

**Published:** 2020-07-10

**Authors:** Xuejiao Liu, Panpan Meng, Guiyan Yang, Mengyan Zhang, Shaobing Peng, Mei Zhi Zhai

**Affiliations:** Laboratory of Walnut Research Center, College of Forestry, Northwest A & F University, Yangling, 712100 Shaanxi China

**Keywords:** Heat stress transcription factors (HSFs), Multiple alignments, Phylogenetic analysis, Motif distribution, Expression profiles

## Abstract

**Background:**

Walnut (*Juglans regia*) is an important tree cultivated worldwide and is exposed to a series of both abiotic and biotic stress during their life-cycles. The heat stress transcription factors (HSFs) play a crucial role in plant response to various stresses by regulating the expression of stress-responsive genes. *HSF* genes are classified into 3 classes: *HSFA*, *HSFB*, and *HSFC*. *HSFA* gene has transcriptional activation function and is the main regulator of high temperature-induced gene expression. *HSFB* gene negatively regulates plant resistance to drought and NaCl. And *HSFC* gene may be involved in plant response to various stresses. There are some reports about the HSF family in herbaceous plants, however, there are no reports about the HSFs in walnut.

**Result:**

In this study, based on the complete genome sequencing of walnut, the bioinformatics method was used and 29 *HSF* genes were identified. These *HSF*s covered 18 *HSFA*, 9 *HSFB*, and 2 *HSFC* genes. Phylogenetic analysis of these HSF proteins along with those from *Arabidopsis thaliana* showed that the HSFs in the two species are closely related to each other and have different evolutionary processes. The distribution of conserved motifs and the sequence analysis of *HSF* genes family indicated that the members of the walnut HSFs are highly conserved. Quantitative Real-Time PCR (qRT-PCR) analysis revealed that the most of walnut *HSFs* were expressed in the walnut varieties of ‘Qingxiang’ and ‘Xiangling’ under high temperature (HT), high salt and drought stress, and some *JrHSFs* expression pattern are different between the two varieties.

**Conclusion:**

The complex *HSF* genes family from walnut was confirmed by genome-wide identification, evolutionary exploration, sequence characterization and expression analysis. This research provides useful information for future studies on the function of the *HSF* genes and molecular mechanism in plant stress response.

## Background

Walnut (*Juglans regia*) is an important nut tree cultivated worldwide [1]. In 2017, its planting area was about 489,866 ha, and the output was about 1,925,403 tons in China (FAO, http://www.fao.org/faostat/en/#data/QC/visualize). However, the walnut is suffering from both abiotic (e.g., high temperature, drought, high salt, chilling.) and biotic (e.g., pathogenic microorganisms and pests) stresses during its life-cycle. In recent years, as “greenhouse effect” has intensified all around the world, high temperature (HT) have reduced the yield of most agricultural and forestry crops to some degree, including the walnut plants. It is generally believed that with a temperature 10 °C to 15 °C higher than the ambient temperature, plants would have a heat shock response (HSR) and obtain a heat resistance quickly within a few hours to withstand the HT which may be lethal [2]. Meanwhile, in China, walnuts are mainly planted in arid and semi-arid regions, where are drought and less rainfall in spring and summer, and precipitation is unevenly distributed. Therefore, moisture is one of the key factors that affect the growth and development of walnuts, as well as the yield and quality of nuts [3]. Moreover, soil salinization and secondary salinization deserve attention. Excessive salt can cause imbalance in osmotic regulation of plants, and excessive accumulation of Na^+^ can also cause ion toxicity. Therefore, the effect of the HT, drought and salt stress on the growth and development of the walnut cannot be ignored.

Gene expression changes triggered by various abiotic stresses are important mechanisms that enable plants to respond and adapt to adverse conditions and thus ensure survival [4]. Therefore, the possible impact of heat, salt and drought stress on walnuts and its molecular mechanisms have been widely surveyed in recent years [5]. Heat stress transcription factors (HSFs) is a protein with transcriptional regulatory activity that responds to a variety of stresses [6]. HSF proteins have five typical structural features: a highly conserved DNA-binding domain (DBD) at the N-terminus, an oligomerization domain (OD or HR-A/B), a nuclear localization signal (NLS), a nuclear export signal (NES) and a C-terminal domain (CTD) at the C-terminus [7]. The DBD domain of HSFs can accurately recognize the heat shock element (HSE: 5′-AGAAnnTTCT-3′; n: any base) that located in the upstream promoter region of *heat shock proteins* (*HSPs*) genes and induce the transcription of *HSP* genes [8, 9]. Depending on the number of amino acids that inserted between the A and B segments of the HR-A/B region, *HSF* genes are classified into 3 classes: *HSFA*, *HSFB*, and *HSFC*. The structure of the class *HSFB* is relatively simple and has no amino acid inserted between the A and B segments, whereas the classes *HSFA* and *HSFC* have 21 and 7 amino acids inserted between the A and B segments, respectively [10]. The CTD of most class *HSFA* is acidic and contains short peptide motifs (acidic amino acid residues, AHA: Activator motifs) with central Trp or Phe residues [11, 12]. The AHA motifs are essential for activation function, and a similar motif has also been identified as part of the activation region of transcription factors (TFs) in mammalian and yeast (*Saccharomyces cerevisiae*) [12, 13]*. HSFB* and *HSFC* lack the AHA motifs; and therefore, they are considered to have no transcriptional activation function. Under normal conditions, *HSF*s exists in the cytoplasm and nucleoplasm without the activity binding to DNA. HSP70 (or HSP90 and multi-companion complex) interacts with HSFs to make HSFs in a passivated monomer state. However, abnormal proteins will be produced during heat shock and then HSPs can be deprived from HSFs and release HSFs, further, HSFs in the nucleus will be assembled into a trimer that binds to the thermal response element at the 5′ end of the heat shock to induce transcription [14].

Although the adversity response function of *HSF* genes is not well understood in most plants, the information about the *HSFs* has accumulated in *A. thaliana* and *Lycopersicon esculentum*. For example, in *L. esculentum*, *HSFA1a* has been found to be a major regulator for the induction of heat-resistant genes and synthesis of *HSFA2* and *HSFB1* [15, 16]; The expression of *HSFA1*, *HSFA2* and *HSFB1* were affected by salicylic acid (SA) under heat shock conditions [17]. In *A. thaliana*, *HSF1*, *HSF3*, *HSFA2* and *HSFA3* are related to heat tolerance, and *HSFA2* is the most strongly induced one by heat; overexpression of *HSFA2* not only enhances plants with basic and heat resistance, but also improves the tolerance of root callus; When osmotic stress occurs, *HSFA2* mutations lead to a significant reduction in basic heat tolerance and antioxidant capacity [18]. Due to the extensive multifaceted roles in anti-stress response, the *HSFs* has recently attracted broad attentions. However, there were few reports on walnut *HSFs*. Considering that abiotic stress causes a significant reduction in walnut yield, and *HSFs* plays a non-negligible role in plant stress resistance, a better understanding of the function of walnut *HSF* genes is important. In this study, the walnut *HSFs* was identified and analyzed according to the released genome [1]. Phylogenetic tree analysis revealed that the evolutionary relationships of HSFs between walnut and *A. thaliana* are different. Quantitative Real-Time PCR (qRT-PCR) analysis provided a solid basis for further functional characterization of the *HSF* genes. In addition, the results may provide vital information for understanding the walnut adversity adaptation mechanism, which will benefit for walnut industry.

## Results

### Genome-wide identification and chromosomal locations of walnut *HSF* genes

Total 33 candidate *HSF* genes were identified by BLAST and HMMER methods. Among the 33 candidates, 4 sequences were repeated and abandoned. Finally, 29 walnut *HSF* genes were confirmed and named from *JrHSF01* to *JrHSF29*. The molecular weight of these *HSF* proteins is between 14.43 kDa and 65.42 kDa, consisting with 128 ~ 505 amino acid residues. The theoretical PIs of these *JrHSF*s are 2.12 ~ 9.28 (Table 1).

**Table 1:** The *HSF* genes in *J. regia*

Gene	Accession No.	Gene ID	Chromos-ome	Number of amino acids/aa	Molecular weight/kDa	Theoretical PI
*JrHSF01*	XP_018829016.1	LOC108997276	Chr3S	500	55.64	5.12
*JrHSF02*	XP_018811267.1	LOC108983931	Chr1D	277	30.62	5.26
*JrHSF03*	XP_018845450.1	LOC109009449	Chr2D	480	53.69	4.89
*JrHSF04*	XP_018845303.1	LOC109009313	Chr2D	471	52.78	4.84
*JrHSF05*	XP_018829017	LOC108997276	Chr3S	500	55.64	2.12
*JrHSF06*	XP_018856444	LOC109018727	Chr4D	363	41.39	5.69
*JrHSF07*	XP_018830717	LOC108998591	Chr2D	300	33.45	6.26
*JrHSF08*	XP_018839407	LOC109005079	Chr1S	368	42.22	4.96
*JrHSF09*	XP_018849985	LOC109012680	Chr5D	390	44.47	5.26
*JrHSF10*	XP_018844061.1	LOC109008434	Chr1D	505	65.42	5.75
*JrHSF11*	XP_018818650	LOC108989484	Chr1D	321	37.37	5.58
*JrHSF12*	XP_018847363	LOC109010870	Chr3D	128	14.43	8.8
*JrHSF13*	XP_018848541.1	LOC109011701	Chr1S	490	54.58	5.58
*JrHSF14*	XP_018805575.1	LOC108979361	Chr2D	336	38	5.49
*JrHSF15*	XP_018813737.1	LOC108985770	Chr1S	277	30.71	6.23
*JrHSF16*	XP_018838842.1	LOC109004663	Chr2S	332	37.52	5.59
*JrHSF17*	XP_018811948.1	LOC108984436	Chr6D	344	37.28	4.82
*JrHSF18*	XP_018847277.1	LOC109010817	Chr3D	505	55.39	5.02
*JrHSF19*	XP_018820155	LOC108990606	Chr5D	206	23.63	9.28
*JrHSF20*	XP_018822295	LOC108992254	Chr8D	363	40.65	8.16
*JrHSF21*	XP_018839503	LOC109005155	Chr5S	503	56.92	5.14
*JrHSF22*	XP_018848321.1	LOC109011524	Chr5S	208	24.25	8.5
*JrHSF23*	XP_018805848.1	LOC108979602	Chr5S	390	44.34	5.05
*JrHSF24*	XP_018818420.1	LOC108989320	Chr5D	250	28.54	6.97
*JrHSF25*	XP_018855724.1	LOC109017997	Chr7S	287	33.17	6.46
*JrHSF26*	XP_018836499.1	LOC109003006	Chr3D	351	40.31	4.78
*JrHSF27*	XP_018809115.1	LOC108982254	Chr6S	497	55.65	4.84
*JrHSF28*	XP_018858526.1	LOC109020508	Chr8D	383	42.96	4.69
*JrHSF29*	XP_018811530.1	LOC108984137	Chr6S	489	54.72	4.84

**Fig. 1:**
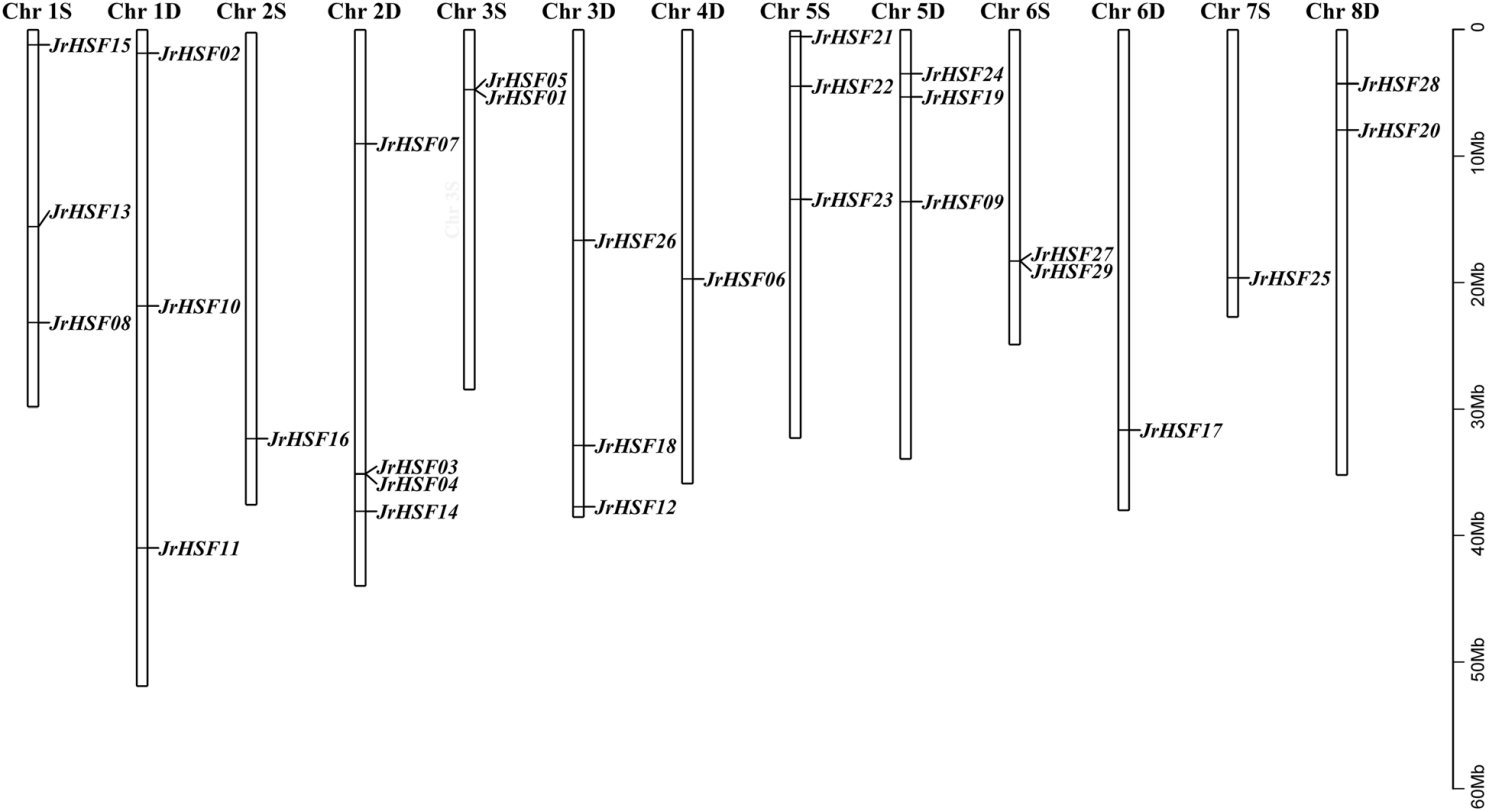
Distribution of the *JrHSF* genes on pseudo chromosomes of *J. regia*. The scale on the right is in million bases (Mb). D: Dominant; S: Subdominant

These 29 *JrHSF* genes were located on 13 chromosomes of *J. regia*, while the chromosomes 4S, 7D and 8S do not contain any *JrHSF* gene. There are 4 *JrHSF* genes mapped on the chromosome 2D, which contains the most number of *JrHSF* genes. The chromosomes 2S, 4D, 6D and 7S each contain only 1 *JrHSF* gene (Fig. 1).

### The conservative domains of *JrHSFs*

**Fig. 2:**
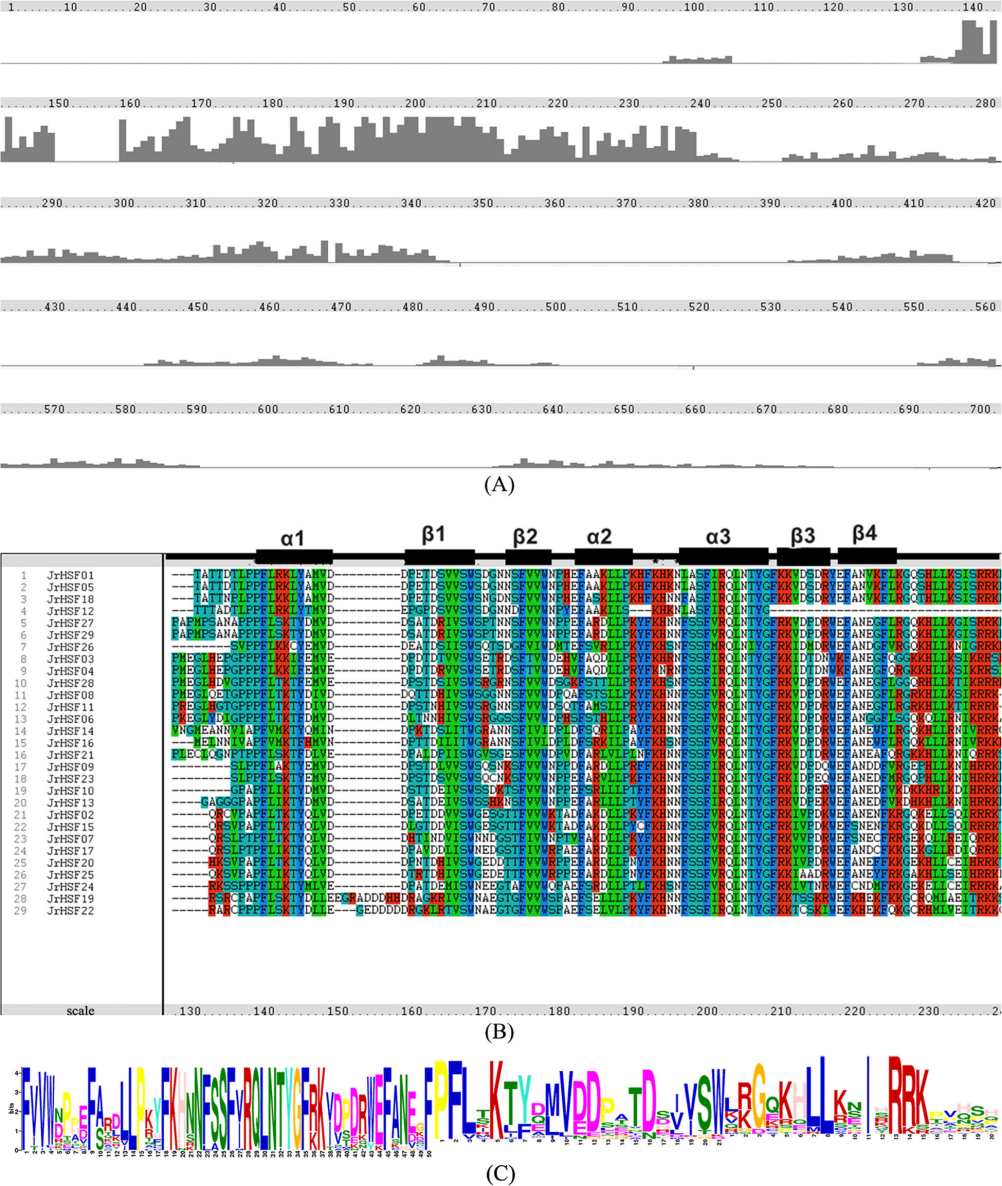
Multiple sequences alignment of JrHSFs. **a** Comparison of amino acid sequences of 29 HSFs in *J. regia*. **b** Multiple sequence alignment of the DBDs of JrHSF proteins. **c** The logo map of JrHSF DBDs

The *JrHSFs* protein sequences were aligned and the result showed that the DBD domain exists in all *JrHSF* sequences and is highly conserved. The number of amino acid residues is from 94 (*JrHSF12*) to 103 (*JrHSF19*) (Fig. 2a). However, there are different degrees of insertion or deletion in these proteins. For example, 9 amino acids are inserted between α1 and β1 in *JrHSF19*, 6 amino acid sequences are inserted between α1 and β1 in *JrHSF22*, however, 36 amino acid residues are lacked in *JrHSF12*, who delete 3 amino acid residues between α2 and α3, and 33 amino acid residues between β3 and β4 (Fig. 2b).

Sequence logos were constructed using WebLogo program and showed that the HSF domain is highly-conserved with 100% of amino acids sequence identity at sites of 1, 9, 14, 15, 18, 22, 25, 26, 28, 29, 30, 31, 33, 34, 35, 45, 50, and so on (Fig. 2c).

### Evolutionary relationship of the *JrHSF* genes

**Fig. 3:**
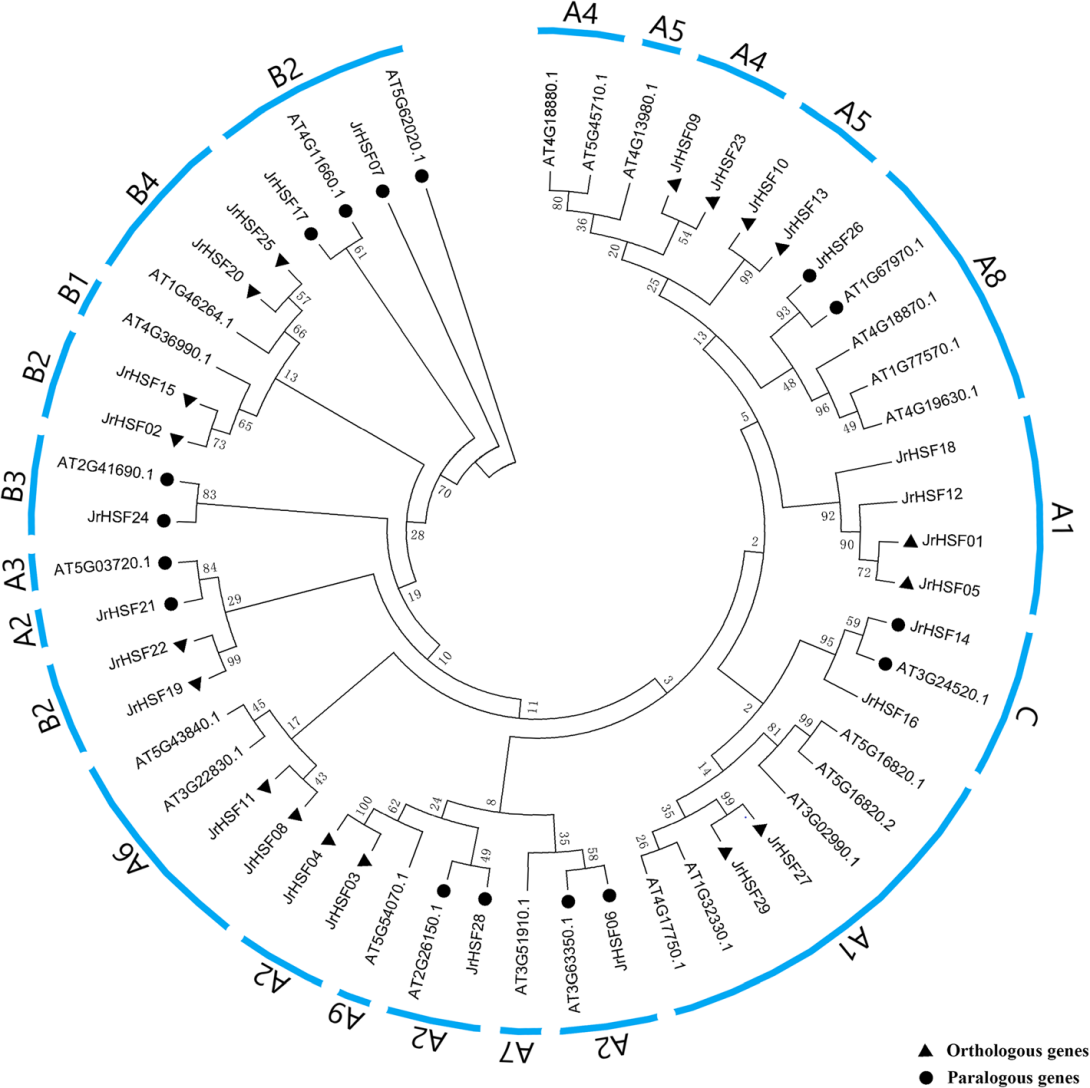
Phylogenetic tree analysis of the *HSFs* from *J. regia* (Jr) and *Arabidopsis* (AT). Orthologous and paralogous genes were indicated by circle and roundness, respectively

An un-rooted phylogenetic tree relating to the evolutionary relationship between the *HSFs* from the walnut and *Arabidopsis* was constructed (Fig. 3). According to the classification of *A. thaliana*, the *HSFs* of these two plants was divided into three groups: group A contains 18 *JrHSFs*, group B contains 9 *JrHSFs*, and group C contains 2 *JrHSFs*. The group A was further divided into 9 subgroups (A1 to A9), of which A5 contains only AT4G13980 (*HSFA5*), A7 includes only AT3G51910 (*HSFA7*), and A9 covers only AT5G54070 (*HSFA9*). Meanwhile, no Orthologous and paralogous *HSF* genes from walnut were found in the above three subgroups, suggesting that gene deletions may have occurred during evolution. The group B was divided into four subgroups (B1 to B4), of which B1 contains only AT4G36990 (*HSFB1*) with no homologous from. The group C was not further divided (Fig. 3).

### Conservative motif distribution and sequence feature of the *JrHSFs*

**Fig. 4:**
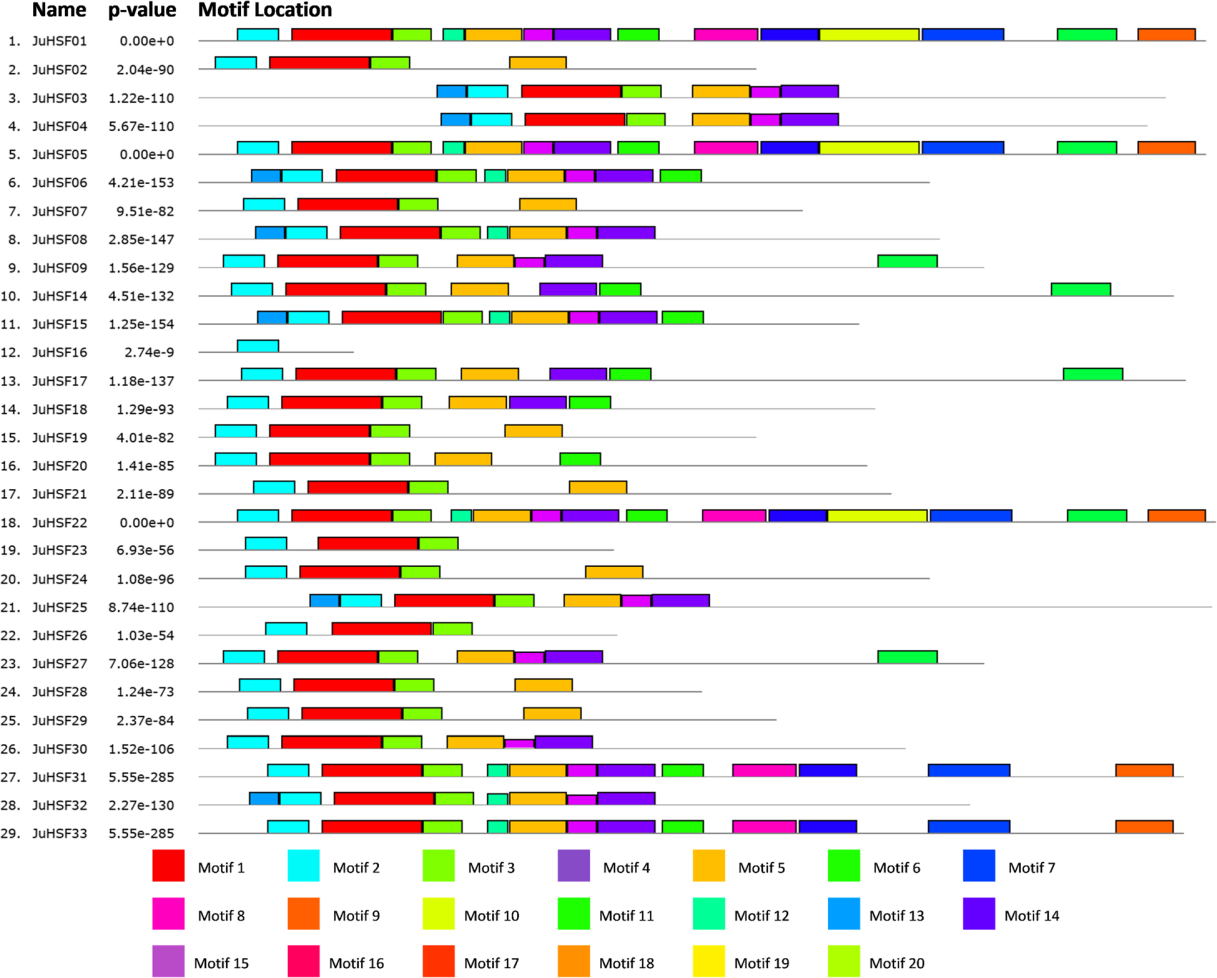
Distribution of the conserved motifs in *JrHSFs*

The MEME was used to analyze the motifs in the *JrHSFs* and their basic information (Table 2 and Fig. 4). The results showed that there are 20 different conserved motifs (including 11 ~ 50 amino acids) in these 29 *JrHSF*s, and each *JrHSF* include 2 ~ 16 conserved motifs (Table 2, Fig. 4). Among 29 *JrHSF*s, *JrHSF01*, *JrHSF05* and *JrHSF22* have the most number of conserved motifs, while *JrHSF16* contains only 2 conserved motifs; *JrHSF23*, *JrHSF24* and *JrHSF26* contain 3 conserved motifs; *JrHSF07*, *JrHSF21*, *JrHSF28* and *JrHSF29* contain 4 conserved motifs, respectively. Furthermore, there are 4 motifs (Motif1, Motif2, Motif3 and Motif4) are completely conserved in these 29 *JrHSFs*. Most of the 29 *JrHSFs* have Motif1, Motif2 and Motif3. The Motif1 (FVVWBPPEFARDLLPKYFKHNNFSSFVRQLNTYGFRKVDPDRWEFANEGF) is located in β2-β4. The Motif2 (PFLTKTYDMVDDPATDSIVSW) is located in α1-β1. The Motif3 is dispersed in the DBD and is highly conserved (Fig. 4). The Motifs 1–3 represent the DBD domain. Therefore, it is concluded that the members of the *JrHSFs* are highly conserved.

**Table 2:** Motif sequences identified by MEME tool

Motif	Number of amino acids	Best possible match
Motif1	50	FVVWBPPEFARDLLPKYFKHNNFSSFVRQLNTYGFRKVDPDRWEFANEGF
Motif2	21	PFLTKTYDMVDDPATDSIVSW
Motif3	20	LRGQKHLLKNIHRRKPVHSH
Motif4	29	MEQRQQQMMSFLAKAMQNPGFJAQLVQQQ
Motif5	29	FGLEEEIERLKRDKNVLMQELVKLRQQQZ
Motif6	30	APVPPGINDTFWEQFLTETPGTSDADEISS
Motif7	41	EETILPEFSEJQGIMPESTAEIPDMNYAGSETGNASYVDPM
Motif8	32	PSMNEAAKALLRQILKMBGSPRVEPLMBNPGA
Motif9	29	HGLZQGKKNGWDKIQHMDKLTEQMGLLAS
Motif10	50	PISAELFMPAEPEFPISSPSTANSDIQSSSYAMPDHAIEAQFPNLDVYNS
Motif11	21	NRRITAGNKKRRLPIEEESES
Motif12	11	SSLGACVEVGK
Motif13	15	PPPQPMEGLHETGPP
Motif14	29	JFDDAPSTNAFDSGSSTNRVSGVTLSEVP
Motif15	15	ATDHQLQAMEQRLQG
Motif16	11	NAPDGQIVKYQ
Motif17	15	MADVNDAGSSTTATT
Motif18	50	VNGSLPIEIDYISPDADIDLFLSDPNFWDDLVQSPVPEDIESNSVQGMSK
Motif19	29	KFGSDQEDLIVKQGDCGGSRGGLVEQAGG
Motif20	48	KQQKRELDGEEFVKRRRLLASHGREKAIDKIHRINCRNQVPGGLVTTL

### Expression of the *JrHSFs* in the walnut

**Fig. 5:**
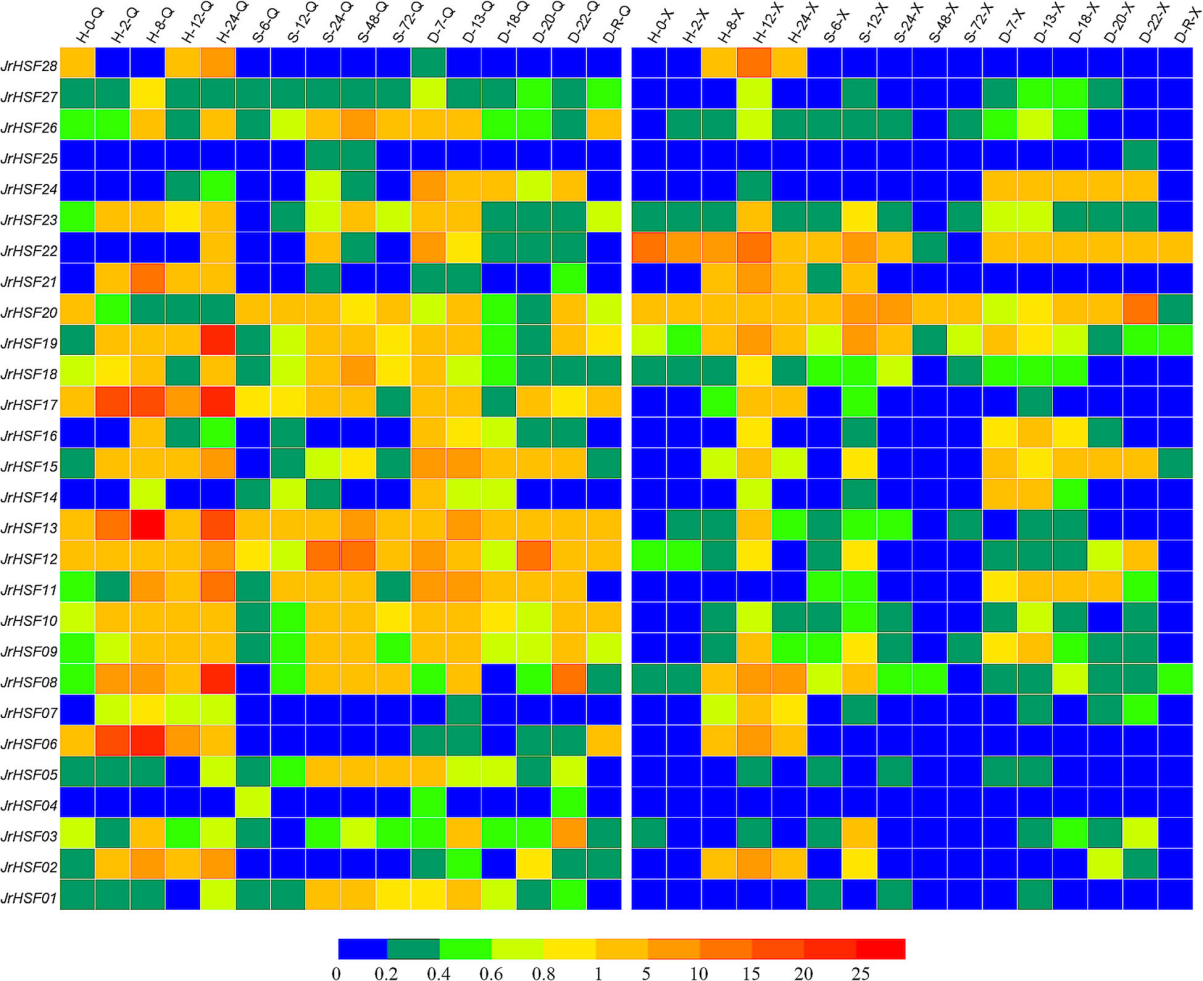
Expression patterns of *JrHSFs* in ‘Qingxiang’ and ‘Xiangling’. H-0-Q / H-0-X ~ H-24-Q / H-24-X: 0, 2, 8, 12, 24 h after high temperature stress treatments in‘Qingxiang’ / ‘Xiangling’. S-6-Q / S-6-X ~ S-72-Q / S-72-X: 6, 12, 24, 48, 72 h after high salt stress treatments. D-7-Q / D-7-X ~ D-22-Q / D-22-X: 7, 13, 18, 20, 22 d after drought stress treatments. D-R-Q / D-R-X: Rehydration. Details of the experimental conditions were provided in Table S2. Log2 based fold changes were used to create the heatmap. Differences in gene expression changes were shown in color as per the lower scale

The expression of *JrHSFs* was analyzed in the leaves of the ‘Qingxiang’ and ‘Xiangling’ using qRT-PCR under drought, HT and high salt stresses. The results showed that all *JrHSFs* were expressed under these stresses with different expression patterns (Fig. 5).

In **‘Qingxiang’.** (1) Under drought stress, the expression of eight *JrHSFs* (*JrHSF24*, *JrHSF13*, *JrHSF19*, *JrHSF15*, *JrHSF11*, *JrHSF22* and *JrHSF09*) were increased at 7 d and then decreased at 22 d; the expression levels of *JrHSF08*, *JrHSF20* and *JrHSF03* were enhanced at 13 d; five *JrHSFs* (*JrHSF16*, *JrHSF24*, *JrHSF15*, *JrHSF11* and *JrHSF05*) maintained high expression after 7 d, but low transcription after rehydration. (2) After heat stress, the transcription of *JrHSF21*, *JrHSF13* and *JrHSF06* reached to peak level at 8 h; five other *JrHSFs* (*JrHSF28*, *JrHSF17*, *JrHSF19*, *JrHSF08* and *JrHSF11*) increased to maximum level at 24 h; and eight *JrHSFs* (*JrHSF21*, *JrHSF13*, *JrHSF06*, *JrHSF17*, *JrHSF02*, *JrHSF19*, *JrHSF08* and *JrHSF15*) maintained high expression status after 2 h. (3) Under salt stress, thirteen *JrHSFs* (*JrHSF13*, *JrHSF17*, *JrHSF19*, *JrHSF8*, *JrHSF15*, *JrHSF26*, *JrHSF18*, *JrHSF23*, *JrHSF10*, *JrHSF05*, *JrHSF01*, *JrHSF22* and *JrHSF09*) showed the highest abundance at 24 h and then decreased. In summary, the expression of *JrHSF28*, *JrHSF21*, *JrHSF06*, *JrHSF02* and *JrHSF07* under heat stress was significantly higher than that under drought and salt stresses, but the expression patterns of *JrHSF05* and *JrHSF01* was opposite. In addition, *JrHSF13* and *JrHSF17* maintained high expression levels while *JrHSF25*, *JrHSF04* and *JrHSF12* were hardly expressed under above three stresses (Fig. 5).

In **‘Xiangling’.** (1) Under drought stress, the expression level of *JrHSF20* reached to peak level at 22 d, most of the *JrHSFs* were hardly expressed after rehydration. (2) After heat stress, *JrHSF22* maintained a high expression level from 0 h to 24 h; the expression of nine *JrHSFs* (*JrHSF28*, *JrHSF21*, *JrHSF08*, *JrHSF17*, *JrHSF06*, *JrHSF02*, *JrHSF07*, *JrHSF19* and *JrHSF15*) were increased significantly at 8 h. (3) Under salt stress, five *JrHSFs* (*JrHSF22*, *JrHSF21*, *JrHSF08*, *JrHSF19* and *JrHSF20*) reached to maximum value at 12 h, while the others displayed low expression levels. In general, *JrHSF22*, *JrHSF08*, *JrHSF19* and *JrHSF20* maintained high expression levels while *JrHSF25* and *JrHSF04* were hardly expressed under above three stresses (Fig. 5).

In short, most of *JrHSFs* responded to HT, high salt and drought stresses in walnut, the expression pattern of most *JrHSFs* was different between ‘Qingxiang’ and ‘Xiangling’.

## Discussion

### The walnut *HSF* genes displayed diverse characters

A phylogenetic tree was constructed with 29 HSF proteins from *J. regia* and 25 HSF proteins from *A. thaliana* (Fig. 3). Nine pairs of orthologous genes and eight pairs of paralogous genes were found, and the paralogous genes of *A. thaliana HSFs* AT4G18870 and AT5G54070 were not found in walnut, indicating that most *HSF* members are specific for reproductive isolation in different species. This phenomenon has also been found in other plant gene families. In *Arabidopsis* and rice (*Oryza sativa*), for instance, most *wall-associated kinase* (*WAK*) genes have species-specific expansion [19]. The lineage-specific divergence of *nucleotide binding site–leucine-rich repeat* (*NBS–LRR*) genes may occur to enable plant response to pathogens unique to each species [20]. *Small auxin-up RNAs* (*SAURs*) genes were clustered in species-specific distinct clades and expanded in a species-specific manner [21]. Moreover, Duan et al. [5] believed that *HSFC* may have different functions in the wheat (*Triticum aestivum*), because *TaHsfC3* has no orthologous genes in the rice, *Arabidopsis* and maize (*Z. mays*) *HSF* families. In grape (*Vitis vinifera*), *Homeobox* (*HB*) genes were not found in three subgroups (PLINC, NDX or LDof) of *Arabidopsis*, Li et al. [22] revealed that *HB* genes may have been deleted during evolution. Although the apple (*Malus domestica*) *WAKY* genes has a small number of mutations compared to the *Arabidopsis WAKY* genes, it also shows that the plant *WAKY* genes family evolved in diversity [23]. All these findings suggested that they had undergone varying degrees of species-specific changes, and these *JrHSFs* have abundant characters.

### *JrHSFs* gene expression patterns responded to abiotic stresses implied multiple roles

*JrHSFs* displayed various expression patterns in one walnut cultivar exposed to HT, drought and high salt stresses, and also showed diverse transcription profiles in ‘Qingxiang’ and ‘Xiangling’ under each of above three treatments (Fig. 5), for instance, under HT stress, in ‘Xiangling’, *JrHSF13* had the largest change in expression, while in ‘Qingxiang’, *JrHSF22* showed the largest change in transcription; *JrHSF02*, *JrHSF06*, *JrHSF08*, *JrHSF17*, *JrHSF19* and *JrHSF21* all revealed changing expression profiles in either ‘Qingxiang’ or ‘Xiangling’; The expression levels of *JrHSF04*, *JrHSF12* and *JrHSF25* were generally low. Interestingly, different types of *JrHSFs* were expressed differently, which is similar to other species. For example, *HSFA1* members of *JrHSF01*, *JrHSF05*, *JrHSF12*, *JrHSF18* and *JrHSF27* show obvious expression under salt stress, which is consistent with the findings observed by Duan et al. [5], who believed that the mutant strain of *HSFA1a* was highly sensitive to salt stress, and all *HSFA1* were involved in osmotic stress response. *JrHSF02* belongs to *HSFB* and had a higher expression level under heat stress, while hardly expressed under drought and salt stresses. This result is consistent with the findings of Li et al. [24] that the rice *OsHSF2b* gene negatively regulates plant resistance to drought and salt; and the negative regulation of *OsHSF2b* is mediated by its C-terminal DBD domain. *HSFCs* including *JrHSF14* and *JrHSF16* have relatively low expression levels under three stresses, and the function of *HSFC* needs further study. These results indicated potential abundant response mechanism for different cultivars exposed to the same stimulus and for different *HSF* members in the same variety under a stress.

Besides, the response of *JrHSF08* under HT stress showed an up-regulation trend with treatment time prolong in both varieties (Fig. 5). *JrHSF08* belongs to *HSFA6*, indicating that *HSFA* may play a key role in resisting abiotic stress, which is consistent with the findings of Liu et al. [25]. *JrHSF04* and *JrHSF25* were not significant in response to stress. This result may indicate that these genes are less abundant in walnuts. *JrHSF13*, *JrHSF12*, *JrHSF11*, *JrHSF10*, and *JrHSF9* were significantly different in ‘Qingxiang’ and ‘Xiangling’, and their expression levels in ‘Qingxiang’ were higher than in ‘Xiangling’. The reason why late mature walnut is more resistant than precocious walnut may be related to these genes [26]. *JrHSF22* had a stronger response to drought and HT stress in ‘Xiangling’ than in ‘Qingxiang’, but it was rarely expressed in ‘Qingxiang’ under salt stress. *JrHSF22* belongs to *HSFB2*, and *HSFB* may negatively regulate plants to respond to salt stress, which is consistent with the results from Scharf et al. [8]. Meanwhile, the time when the gene expression reaches to the peak was various, implying the initiation of the *JrHSFs* expression may be different. All these findings suggested that the concrete role of every *HSF* gene is plant-specific to special conditions.

Moreover, most of the *JrHSFs* show higher expression levels under the three stresses in ‘Qingxiang’ than those in ‘Xiangling’. This result was also observed in grape varieties ‘Jingxiu’ and ‘Tangwei’. What’s more, Liu et al. [25] used this result to predict the potential stress resistant ability of ‘Jingxiu’ and ‘Tangwei’. Therefore, we may also conclude that ‘Qingxiang’ is more resistant to abiotic stress than ‘Xiangling’, which further confirmed the fact that ‘Qingxiang’ is more tolerant to adversity than ‘Xiangling’. Meanwhile, HSF genes may be functioned as resistance markers to distinguish different plants responding to the same stress.

### Potential regulation of *HSFs* in heat temperature, drought and salt stresses

Plants can resist the HT stress by producing hormones and osmotic substances. For example, SA pathway can mediate the system-acquired heat resistance (SAR). SA promotes the production of HSPs, and also effectively removes H_2_O_2_ from cucumber [27]. HSPs can increase the denaturation temperature of proteins in plants to protect them from HT damage, and repair the damaged proteins to resist the high temperatures. The expression of HSPs is mainly regulated by the HSFs at the transcriptional level [28]. Like the mammalian HSFs, in plants, the binding of a chaperone to a misfolded protein forces the HSFs to be released to activate expression of the thermosensitive genes. Meanwhile, HSF is a highly conserved central regulator of the HSR in eukaryotes [29]. For example, *HSFA2* is accumulated under the HT stress and functioned on signal amplification [30]. *HSFB2b* gene is required for obtaining heat resistance in plants [24]. Rice *OsHSFC1a* can respond to early HS treatment [29].

Moreover, the role of the HSF signaling pathway is not only limited to thermal stress responses, but also involves in a variety of stresses such as cold, infiltration, drought, salt, ultraviolet light, oxidation and pathogen infection [31]. In the field, the drought stress usually occurs simultaneously with the HT stress. *A. thaliana* Galactinol synthase (*GolS1*) is an important compound produced by plants in response to the drought stress, whose expression is regulated by HT; meanwhile, target *GolS1* could mediate the HSFs in plant response to the drought stress [32]. In addition, the HSFs can also act on the downstream drought response element binding factors (DREB2A and DREB2C) to improve the expression of some drought-stress-response genes [33]. Similarly, when subjected to the salt stress, plants are actually subjected to the osmotic stress. This osmotic stress signal can cause the accumulation of abscisic acid (ABA), which further induces the adaptive response of plants [34]. In *A. thaliana*, the promoter of *AtHSFA6a* contains two AREs (ABA response elements) and binds to three ABA-responsive TFs in vitro; the plants overexpressing *AtHSFA6a* exhibit both salt and drought tolerance [28]. Peng et al. [35] showed that in grapes the expression of the *HSFs* is induced by the pathogen infection, HTs and drought, suggesting that the *HSFs* are associated with both biotic and abiotic stresses in grapes. Interestingly, salt damage, drought stress, and other osmotic stress lead to ROS (Reactive Oxygen Species) accumulation and cell wall changes. Cell wall contains protein and Ca^2+^ ions [36]. The salt stress can also cause loss of Ca^2+^ in the cell wall. Ca^2+^ and ROS are key factors in the process of evoking the HSR [37–40]. Therefore, it draws our notion that *JrHSF* genes response to stresses may involve in HSR and ABA signal pathway. To verify this point, further research is needed. Although it is not clear further principles of the *HSF* genes responding to the HT, salt and drought stresses, this research has shown that the *HSF* genes do participate in resistance to the HT, drought and salt stresses in the walnut.

## Conclusions

In this study, 29 *JrHSFs* were identified and their distribution on chromosomes, gene structure, conserved motifs, and evolutionary relationships were analyzed, and found that the *JrHSFs* are highly conserved and displayed different evolutionary processes from *AtHSFs*. *JrHSF17*, *JrHSF13*, *JrHSF12*, *JrHSF11*, *JrHSF10*, and *JrHSF9* were significantly induced and different in both cultivars, implying the necessary attention and further exploration. The various expression of *JrHSFs* in ‘Qingxiang’ and ‘Xiangling’, suggested that *JrHSFs* may play an important role in plant resistance to high temperature, drought and salt stresses. This research provides useful information for future studies on the function and response mechanism of *JrHSF* genes.

## Methods

### Plant materials

#### Seedling preparation

The tissues used in this experiment were collected from 2-year-old *J. regia* varieties ‘Qingxiang’ (a late bearing walnut variety) and ‘Xiangling’ (a precocious walnut variety), which were preserved in a plant factory of Northwest Agriculture & Forestry University (our lab, in Yangling, Shaanxi province, China) and stored in the Liaoning Economic Forest Research Institute (in Dalian, Liaoning province, China). New branches were obtained and grafted into walnut rootstocks (*J. regia*) which were planted in the mixed turf peat and sand (2:1 v/v) in plastic pots in a greenhouse (22 ± 2 °C, relative humidity 70 ± 5%, 14 h light/ 10 h dark). When the height of the walnut seedlings reached to about 55–65 cm, seedlings with similar height and biomass were selected for further experiments. The collected leaf samples were frozen in liquid nitrogen immediately and stored at − 80 °C for RNA extraction [41, 42].

#### High temperature treatment

The selected 24 seedlings (12 ‘Qingxiang’ walnuts and 12 ‘Xiangling’ walnuts) were heated (42 °C) for 2 h in a greenhouse [5]. The leaves were collected at 0, 2, 8, 12 and 24 h after heating in triplicate, respectively. Three seedlings of each variety grew normally at room temperature (25 °C) as a control.

#### Drought stress treatment

First, the soil moisture of the selected 30 seedlings (15 ‘Qingxiang’ walnuts and 15 ‘Xiangling’ walnuts) was maintained at 60% for 7 days, and then the watering of 9 seedling of each variety was stopped to reduce the soil moisture naturally. When soil moisture reached 60% (7 d), 40% (13 d), 20% (18 d), 10% (20 d) and 5% (22 d), the leaves were collected in triplicate, respectively. Three seedlings of each variety were re-watered. Samplings were performed when soil moisture was increased to 60% and kept for 3 days (REW60%). The other 3 seedlings of each variety under normal watering were used as controls. The soil moisture content of each plant was monitored and adjusted by weighing methods [43].

#### NaCl treatment

First, the selected 24 seedlings (12 ‘Qingxiang’ walnuts and 12 ‘Xiangling’ walnuts) were irrigated with NaCl-free ½ Hoagland solution, until the soil surface was covered by a thin layer of water and this was maintained for 72 h. Then, the solution of 9 seedlings of each variety was replaced with ½ Hoagland nutrient solution containing 300 mM NaCl. When the treatment time reached 6, 12, 24, 48 and 72 h, the leaves were collected in triplicate, respectively. The remaining 3 seedlings of each variety were watered with NaCl-free ½ Hoagland solution and used as a control [43, 44].

### Identification and chromosomal locations of the *HSF* genes

The domain architecture of the *HSF* genes was obtained from Pfam database (http://pfam.sanger.ac.uk) [45] and their chromosomal locations in the walnut genome (*Juglans microcarpa* x *Juglans regia*) were downloaded from NCBI database (https://www.ncbi.nlm.nih.gov/genome/?term=txid2249226[orgn) [46]. The walnut *HSF* gene sequences were downloaded from iTAK (http://itak.feilab.net/cgi-bin/itak/index.cgi) database. Then candidate walnut *HSF* coding region gene sequences were obtained by merged using the software UltraEdit, BLAST (blast-2.60) and HMMER searchers [47]. Conserved Domain Search (https://www.ncbi.nlm.nih.gov/Structure/cdd/wrpsb.cgi) was applied to analyze the core domain of the candidate *HSF*s. ExPASy proteomics server (https://web.expasy.org/protparam/) was used to estimate the amino acid number, the molecular weight and the theoretical isoelectric point (PI) [43].

### Multiple alignments and phylogenetic analyses

ClustalX 2.1 was used to align the protein sequences of the *HSF* genes [48]. The protein sequences of the *HSF* genes from *A. thaliana* and the walnut were used to construct a neighbor-joining tree with 1000 bootstrap replications using MEGA6 [49]. The MEME (Multiple Em for Motif Elicitation) tool (http://meme-suite.org/tools/meme) [50] was chosen to analyze the conservative motifs. The amino acid sequence file was saved into the FASTA format and input into the online tool MEME [51]. The parameters were set as follows: the minimum length of the conservative motif is 6, the maximum length is 50, the maximum number of conserved motifs is 20, and the others are default values.

### qRT-PCR analysis

Total RNA was extracted from the leaf tissues of ‘Qingxiang’ and ‘Xingling’ varieties using an improved CTAB method [52]. First-strand cDNA was synthesized from 1 mg DNase-treated total RNA using a mixture of Oligo dT Primer and Random 6 mers (PrimeScript™ RT Master Mix, TaKaRa, Xi’an, Shaanxi, China) and the reaction volume was 10 μL [53]. Gene-specific primers were designed and shown in Table S1. To verify the specificity of the primers, 28 genes of the 2 varieties were amplified by PCR, respectively (Figure S1). qRT-PCR analysis was conducted using TB green (TB Green™ Premix Ex Taq™ II, Takara) with an IQ5 real-time PCR machine (Bio-Rad, Hercules, CA, USA). Each reaction was carried out in triplicate with a reaction volume of 20 μL. Cycling parameters were 95 °C for 30 s, followed by 40 cycles of 95 °C for 5 s and 60 °C for 30 s. For dissociation curve analysis, a program including 95 °C for 15 s, followed by a constant increase from 60 °C to 95 °C, which was included after the PCR cycles. The qRT-PCR contained three biological repeats. The *18S-RNA* gene [41] was used as an internal control. The primer sequences were listed in Table S1. Relative expression levels were analyzed using the IQ5 software and the normalized expression method. The results were evaluated by the 2^−ΔΔCT^ method [54]. Details of the experimental conditions are provided in Table S2.

## Supplementary information

**Additional file 1: Figure S1.** PCR analysis of *JrHSFs* in ‘Qingxiang’ and ‘Xianging’.

**Additional file 2: Table S1.** Gene-specific primers of *JrHSFs* and *18S-RNA* gene.

**Additional file 3: Table S2.** qRT-PCR relative expression data of Fig. 5.

## Data Availability

All data generated or analyzed during this study are included in this published article [and its supplementary information files]. The planting area and yield of Walnut in China in 2017 were obtained from FAO (http://www.fao.org/faostat/en/#data/QC/visualize). The domain architecture of the *HSF* genes was obtained from Pfam database (http://pfam.sanger. ac.uk). The chromosomal locations in the walnut genome (*Juglans microcarpa* x *Juglans regia*) were downloaded from NCBI database (https://www.ncbi.nlm. nih.gov/genome/?term=txid2249226[orgn). The walnut *HSF* gene sequences were downloaded from iTAK (http://itak.feilab.net/cgi-bin/itak/index.cgi) database. Conserved Domain Search (https://www.ncbi.nlm.nih.gov/Structure/cdd/wrpsb.cgi) was applied to analyze the core domain of the candidate *HSF*s. ExPASy proteomics server (https://web.expasy.org/protparam/) was used to estimate the amino acid number, the molecular weight and the theoretical isoelectric point (PI). The MEME (Multiple Em for Motif Elicitation) tool (http://meme-suite.org/tools/meme) was chosen to analyze the conservative motifs.

## Abbreviations

HT: High temperature
HSR: Heat shock response
HSFs: Heat stress transcription factors
DBD: DNA-binding domain
OD or HR-A/B: Oligomerization domain
NLS: Nuclear localization signal
NES: Nuclear export signal
CTD: C-terminal domain
HSE: Heat shock element
HSPs: Heat shock proteins
AHA: Activator motif
TFs: Transcription factors
SA: Salicylic acid
qRT-PCR: quantitative Real-time PCR
PI: Isoelectric point
WAK: Wall-associated kinase
NBS–LRR: Nucleotide binding site–leucine-rich repeat
SAURs: Small auxin-up RNAs
HB: Homeobox
SAR: System-acquired heat resistance
GolS1: Galactinol synthase
ABA: Abscisic acid
AREs: ABA response elements
ROS: Reactive Oxygen Species
