# Supplementary material for: Genome-wide identification and transcript profiles of walnut heat stress transcription factor involved in abiotic stress
Source: BMC Genomics. 2020 Jul 10;21:474. doi: 10.1186/s12864-020-06879-2 (PMC7350716; doi:10.1186/s12864-020-06879-2)
Supplement: Supplementary file 1 — Additional file 1: Figure S1. PCR analysis of JrHSFs in ‘Qingxiang’ and ‘Xianging’. [file 12864_2020_6879_MOESM1_ESM.docx]

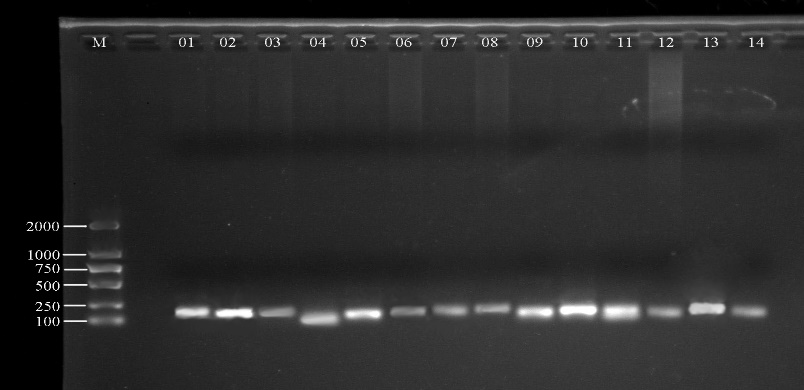


(A)


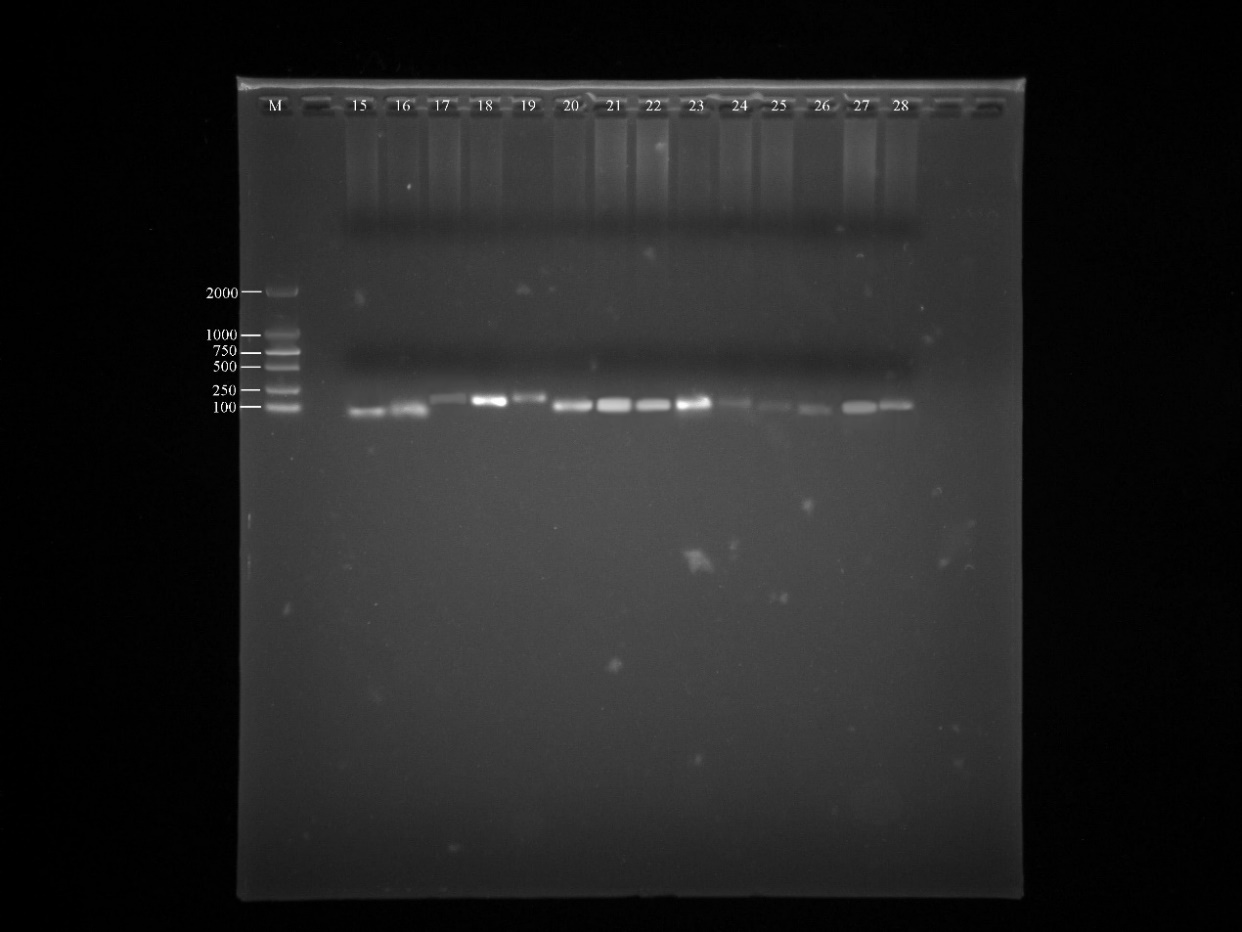


(B)


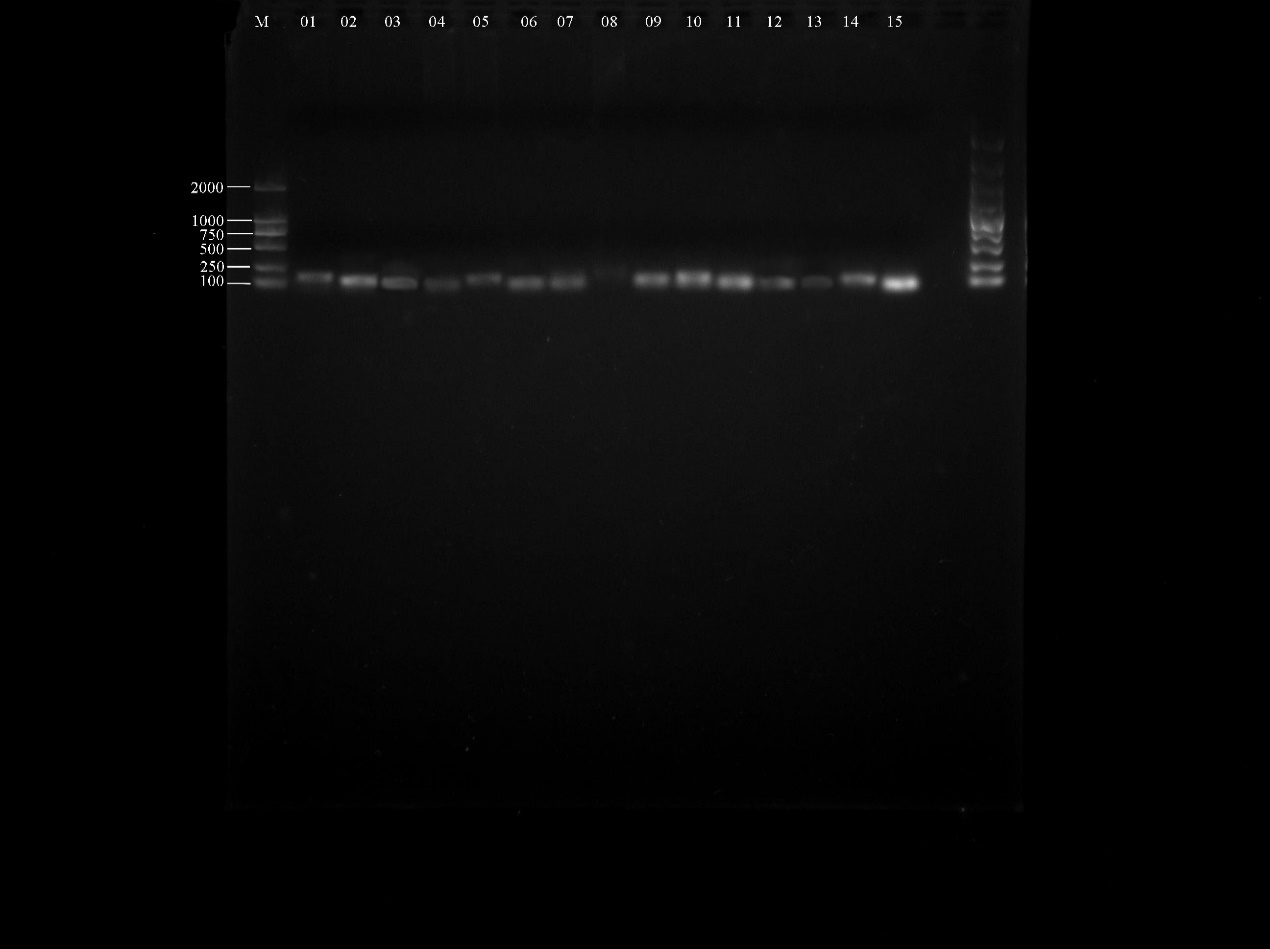


(C)


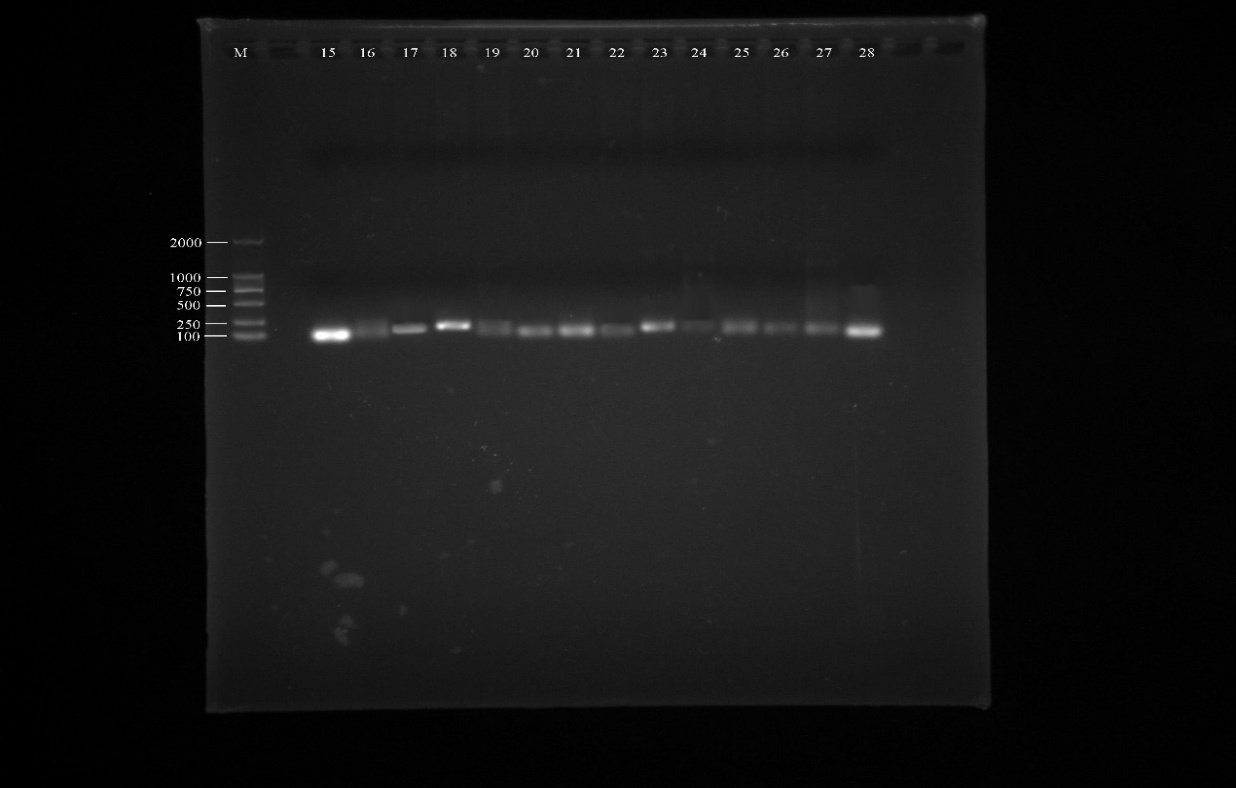


(D)

**Supplemental Figure 1. PCR analysis of *JrHSFs* in ‘Qingxiang’ and ‘Xianging’.** M: DL Marker2000. (A) and (B) 01-28: *JrHSF01*-*JrHSF28* in ‘Qingxiang’. (C) and (D) 01-28: *JrHSF01*-*JrHSF28* in ‘Xiangling’.
