# Supplementary material for: Genome-wide identification and transcript profiles of walnut heat stress transcription factor involved in abiotic stress
Source: BMC Genomics. 2020 Jul 10;21:474. doi: 10.1186/s12864-020-06879-2 (PMC7350716; doi:10.1186/s12864-020-06879-2)
Supplement: Supplementary file 2 — Additional file 2: Table S1. Gene-specific primers of JrHSFs and 18S-RNA gene. [file 12864_2020_6879_MOESM2_ESM.docx]

**Table S1. Gene-specific primers of *JrHSFs* and *18S-RNA* gene.**

| Locus | Forward primer 5’~3’ | Reverse primer 5’~3’ | Size/bp |
| --- | --- | --- | --- |
| *JuHSF01* | CAGCAGAATGAAAGCAATAGGAAC | CCGCAGCAAGGTTTTAGCA | 165 |
| *JuHSF02* | GGAGACGACAACAACAGCACA | ATACCCAGTCAGAAACGCTACCA | 139 |
| *JuHSF03* | TGCTTATGATGTCATGGCAGAGA | TCCTCCTCTAAATCCACCCAAA | 148 |
| *JuHSF04* | GCCTTCTGATGACATTCCTCCT | GTGGTGCGGTGTCGTTTATTT | 116 |
| *JuHSF05* | AGACTCCCCAATCAAGAGGAAAG | CCGCAGCAAGGTTTTAGCA | 117 |
| *JuHSF06* | CCAACGGAGGTTTTCTGAGTG | TGCTTCATTTCTGTCCCCTGT | 265 |
| *JuHSF07* | GCAACAATATCTTCGCTCTGATG | TTCCCTCGCTCTCTTCCTACC | 173 |
| *JuHSF08* | GGTTTCTTAGAGGACGAAAGCATC | TCACGCCTCAACCAATCAAC | 145 |
| *JuHSF09* | CAAGGACAAGGAAGTGCGTCT | CGTGCCTCCGTAACTCCAA | 115 |
| *JuHSF10* | AGATGGAGCAGAGGCAGGAG | CATGTGGCAGTCGTCTTTTCTT | 138 |
| *JuHSF11* | TTCAGAGCTGGAAGAACTTGCTATG | CAGAACCCCTCCTCAAAGTCC | 132 |
| *JuHSF12* | AAATACATTCAACTCGCCGTCA | GAGTTCCATTCCCGCTTCC | 124 |
| *JuHSF13* | TGGAGCAGAGGCAGGAGAA | AACTCTGTGTGCTATGTGAAACCAA | 297 |
| *JuHSF14* | CCGCCTAATGCTCCAGAAA | TTGTTCCCTCTTCTTCGTCCTC | 122 |
| *JuHSF15* | GTGGCATTTCTGACTGGGTATCT | GGTTTCTTCAGTCCCGTTGTTT | 162 |
| *JuHSF16* | TTCCACGTATGATGCTCCAGA | CTCATCCTCATCCTCGGTCTTT | 122 |
| *JuHSF17* | GTAAAGAGGGCGAGGAACGA | ACCACCAACACCTGCCAAC | 273 |
| *JuHSF18* | ACTTCCAGGCATTAACGATACTTTC | TTGTCCCAGTTGTTCTTCTCTCC | 150 |
| *JuHSF19* | GTACTGCTAGTTCAAGCCAATCTCC | ACCAAACAACAAACCCAGTTCC | 165 |
| *JuHSF20* | AGGTTGTTCTTGAGCTTTCGATG | GGTAGGTTTTGGTGAGGAATGG | 111 |
| *JuHSF21* | AGGTTGATGCAGGAGGTTGTT | ACCATTTGCTTCTGTCTTTGCTC | 113 |
| *JuHSF22* | GAACGGGGTTTGTAGTATGGTCTC | GACACTTGGCTCGCACTTCTT | 218 |
| *JuHSF23* | TCGCAGAGGAATGAAGAGGAG | GCAAGACTCCAGCAACAGAGG | 121 |
| *JuHSF24* | GAAGACGTACATGCTGGTGGAG | TATGCTTGAAAAGTGTAGGGAGGAG | 131 |
| *JuHSF25* | TTGGGGTGAAGATGAAACCAC | TGGCGAACAAAACTGGAGAA | 108 |
| *JuHSF26* | TTCCAAACCCGTCCTCACA | GATTTCTTCACCATCACGTTACCA | 119 |
| *JuHSF27* | ACACCTACGGTTTCAGGAAGG | GCTGTGGCTGTTGATGACTATG | 138 |
| *JuHSF28* | GCACAGTAACTTCTCCAGCTTCATT | GTCTTCTCCGCTTGATGGTCTT | 140 |
| *18S-rRNA* | GGTCAATCTTCTCGTTCCCTT | TCGCATTTCGCTACGTTCTT | － |
